# Clinical, Microbiological, Serological and Radiological Profile of Patients With Mild-Moderate and Severe Allergic Bronchopulmonary Aspergillosis (ABPA)

**DOI:** 10.7759/cureus.44662

**Published:** 2023-09-04

**Authors:** Amardeep Singh, Umma Sadia Tomo, Manoj Kumar Dodiya, Pankaj K Singh, Abdul Manan, Inimerla Bhavya, Varsha S Kumar, Irfan A Mir

**Affiliations:** 1 Internal Medicine, Dayanand Medical College and Hospital, Ludhiana, IND; 2 Internal Medicine, Rangpur Community Medical College, Rangpur, BGD; 3 Internal Medicine, Gujarat Cancer Society (GCS) Medical College & Research Institute, Ahmedabad, IND; 4 Emergency Medicine, School of Medical Sciences & Research, Greater Noida, IND; 5 Nephrology, New Cross Hospital, Wolverhampton, GBR; 6 Internal Medicine, School of Medical Sciences & Research, Greater Noida, IND; 7 Internal Medicine, Sapthagiri Institute of Medical Sciences & Research, Bengaluru, IND; 8 Internal Medicine, New Cross Hospital, Wolverhampton, GBR

**Keywords:** spirometry, ige, centrilobular nodule, high attenuation mucus, allergic bronchopulmonary aspergillosis

## Abstract

Objective

Allergic bronchopulmonary aspergillosis (ABPA) is a complex hypersensitivity reaction to Aspergillus antigen mostly Aspergillus fumigatus that occurs almost exclusively in patients with asthma and cystic fibrosis. ABPA is an underdiagnosed and undertreated disease because of its presentation with various grades of severity in asthma patients. Data available regarding the clinical, serological, and radiological profile of ABPA patients is limited due to lack of consensus on diagnostic criteria and treatment guidelines. Thus ABPA is a significant disease, especially in the Indian population where the incidence of allergic diseases like asthma is on the rise.

Methods

This prospective study was conducted in the Department of Pulmonary Medicine at one of the tertiary centers of north India. All consecutive patients diagnosed with allergic bronchopulmonary aspergillosis (ABPA) from 1st January 2017 to 30th September 2017 were included in the study. A total of 67 consecutive patients diagnosed with bronchial asthma were included in the study. The diagnosis of ABPA was based upon either criterion given by Rosenberg and Paterson^ ^or the International Society of Human and Animal Mycology (ISHAM) criteria. Patients diagnosed with ABPA were finally divided into mild, moderate, and severe.

Results

The majority of patients showed an obstructive pattern on spirometry and moderate to severe obstruction was the most common pattern observed among patients who had an obstructive pattern on spirometry. Also, all three patients with the mixed pattern on spirometry had severe disease. Serological analysis revealed that patients in the moderate category had a higher level of absolute eosinophil count (AEC), total IgE, and Aspergillus-specific IgE antibodies, especially in patients who had either high attenuation mucus (HAM) or centrilobular nodules on their high-resolution computed tomography (HRCT) scan.

Conclusion

ABPA is a disease of divergent presentation. We concluded to have alternate or add-on criteria for the classification of ABPA which was not based on the sequelae of chronic inflammatory changes in the lungs.

## Introduction

Allergic bronchopulmonary aspergillosis (ABPA) is a complex hypersensitivity reaction to Aspergillus antigen mostly Aspergillus fumigatus that occurs almost exclusively in patients with asthma and cystic fibrosis [1]. The earlier concept of thick viscid mucus not being cleared off by ciliary action and serving as a ground for the growth of Aspergillus spores present in the environment as the sole reason for the development of ABPA has been disproved as these findings were not coherent with that of patients with a severe form of asthma and cystic fibrosis who didn't always suffer from ABPA. There is a weak correlation between the degree of Aspergillus spores exposure and the development of the disease. Several host characteristics have been identified to explain the selective development of ABPA in a small subset of individuals who are sensitized to the Aspergillus mold. Posterior segments of the upper lobe are most affected in patients with ABPA. Eosinophilic pneumonia and mucoid impaction are common findings in a patient with ABPA compared to severe asthma with fungal sensitization (SAFS) [2]. Despite the similarity of the clinical and serologic presentation of the disease, the parenchymal responses are varied and include eosinophilic pneumonia, broncho-centric granulomatosis, and granulomatous bronchiolitis, exudative bronchiolitis, lipid pneumonia, lymphocytic interstitial pneumonia, desquamative interstitial pneumonia, pulmonary vasculitis, and bronchiolitis obliterans [3].

The diagnosis of ABPA is made in the presence of a constellation of symptoms, sensitization to Aspergillus antigen with radiological abnormalities. For evidence of allergic sensitization, an initial screening test for ABPA is percutaneous or prick test. This test is positive in nearly all ABPA patients and in 20-25% of patients with persistent asthma [4]. The clinico-radiological staging of ABPA is done at the time of diagnosis. With time, this is assessed according to the patient's clinical, serological, and radiological condition as the patient comes for follow-up. In fact, ABPA is an underdiagnosed and undertreated disease because of its presentation with various grades of severity in asthma patients. The data available regarding the clinical, serological, and radiological profile of ABPA patients is limited due to lack of consensus on diagnostic criteria and treatment guidelines. Thus ABPA is a significant disease, especially in the Indian population where the incidence of allergic diseases like asthma is on the rise. Also, there is a paucity of data on the relation of the clinical-radiological profile with serology in various grades of severity of ABPA patients and thus making it a topic of interest [5]. The objective of this study is to find the clinical, microbiological and radiological profiles of patients with mild moderate, and severe ABPA and to study the radiographic-serological association in ABPA patients.

## Materials and methods

This prospective study was conducted in the Department of Pulmonary Medicine at one of the tertiary centers of north India. All consecutive patients diagnosed with allergic bronchopulmonary aspergillosis (ABPA) from 1st January 2017 to 30th September 2017 were included in the study and a total of 67 consecutive patients diagnosed with ABPA with a background history of bronchial asthma were included in the study.

After obtaining informed consent, the data collected from the patients included the social and demographic background of the patient (name, age, address, occupation), presenting complaints, risk factors, examination findings, and appropriate investigations. To support the diagnosis of allergic bronchopulmonary aspergillosis, aspergillus skin test, total IgE levels, fungus culture of sputum, chest X-ray (posterior-anterior view), absolute eosinophil counts (AEC), high-resolution computed tomography (HRCT) scan and spirometry were done. The findings of the chest X-ray and HRCT chest were reported by a single experienced radiologist of the institute. The diagnosis of ABPA was based upon either criterion given by Rosenberg and Paterson [6] or the International Society of Human and Animal Mycology (ISHAM) criteria (Table 1) [7].

**Table 1:** Diagnosis of allergic bronchopulmonary aspergillosis (ABPA) was based upon either criterion given by Rosenberg and Paterson or the International Society of Human and Animal Mycology (ISHAM) criteria

Rosenberg & Paterson CRITERIA	ISHAM CRITERIA
MAJOR (at least 4 must be present)	OBLIGATORY CONDITIONS (both must be present)
Asthma	Aspergillus skin test positivity or detectable IgE levels against Aspergillus fumigatus
Transient pulmonary infiltrates (fleeting opacities)	Elevated total serum IgE concentrations (typically > 1000 IU/ml, but if a patient meets all other criteria, an IgE value < 1000 IU/ml may be acceptable.
Immediate type cutaneous reactivity to Aspergillus antigen	
Elevated total IgE levels (> 1000 IU /dl)	
Specific IgE against A. fumigatus	
Serum-precipitating antibodies against A. fumigatus	
Peripheral blood eosinophilia	
Proximal and central bronchiectasis on CT chest	
MINOR (at least 1 must be present)	OTHER CONDITIONS (at least 2 must be present)
Expectoration of golden brown sputum pugs	Precipitating serum antibodies to A. fumigatus
Late (Arthus type) skin reactivity to A. fumigatus	Radiographic pulmonary opacities consistent with ABPA
	Total eosinophilic count > 500 cells/microlitres in glucocorticoid naive patients

Inclusion criteria and distribution of patients into various grades of severity

Patients diagnosed with ABPA according to the above criteria were further divided into three groups based on the Kumar classification [8].

1. ABPA-S/Mild: Patients with all the criteria positive except central bronchiectasis.

2. ABPA-CB/Moderate: Patients having central bronchiectasis along with the above-discussed criteria.

3. ABPA-CB-ORF/Severe: Patients who have the above features along with other radiological features such as pleural thickening, pleural effusion, mediastinal lymphadenopathy, ground glass appearance, collapse, fibro cavitary lesions, parenchymal scarring, bullae, blebs, aspergilloma, emphysematous changes, multiple cysts, pneumothorax, pulmonary fibrosis.

The patients who were excluded from the study were pregnant females, patients with pulmonary or extrapulmonary tuberculosis, patients with an age of less than 15 or more than 65 years, known cases of cystic fibrosis and patients not giving consent to the study.

This study has been approved by the ethics committee of Dayanand Medical College & Hospital.

Statistical analysis

Statistical analysis was done with Windows Statistical Package for the Social Sciences (SPSS) version 18 (IBM Corp., Armonk, NY, USA). Descriptive statistics were completed using medians and means. Continuous variables were summarized as mean and standard deviation (SD). Categorical variables were summarized as percentages. Comparative analysis between serological parameters of different variants was used based on cross-tabulation. The significance of the relationships in cross-tabulation was tested using the Pearson chi-square test for categorical parameters, while scale variables were compared using the independent samples t-test. P-values (determined by χ2 or Fisher's exact tests) were reported with a value of <0.05 considered significant.

## Results

**Figure 1:**
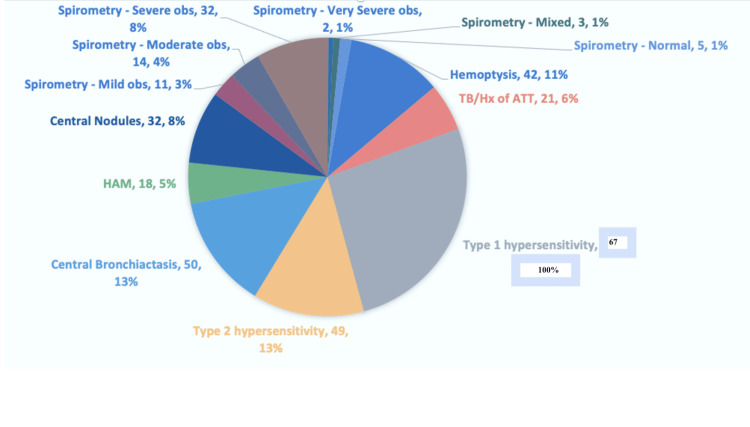
Baseline parameters of the study population. HAM: High Attenuation Mucus, TB: Tuberculosis, Hx: History, ATT: Anti-tubercular therapy, obs: Obstruction

In our study, the mean age of the study population was 36.42 ± 13.59 with around 51% of males. The other baseline characteristics of the study population are shown in Figure 1.

Based on classification by Kumar, out of the 67 patients diagnosed as having ABPA, 37 (55%) were classified as severe (ABPA-CB-ORF), 14 (21%) were moderate (ABPA-CB), and 16 (24%) were mild (ABPA-S). Also, the mean age for the mild category was 43.25 ± 14.2 years, whereas it was 31.50 ± 10.6 years for the moderate category and 35.3 ± 13.4 years for the severe category. Moreover, we documented a statistically significant difference in age of presentation between mild and moderate disease (p=0.017) and between mild and severe disease (p=0.048) with the mild component presenting at a relatively later age. The gender distribution in our study population was 34 males and 33 females. Most of the males either had (10/16) mild or (21/37) severe disease, while most of the females presented with (11/14) moderate disease (p=0.044), (chi-square=6.234), and this difference in the presentation was found to be statistically significant.

The minimum and maximum durations for which patients were diagnosed with bronchial asthma before being diagnosed with ABPA in our study were 2 years and 35 years, respectively. The mean duration of asthma was 8.69 ± 7.68 years in the mild category, 12.5 ± 5.17 years in the moderate and 16.14 ± 6.22 years in the severe category. So, we could say that duration of asthma was significantly higher in the severe form of ABPA (p=0.000) in comparison to the mild form. Also, differences between mild vs. moderate (p=0.01) and moderate vs. severe (p=0.0075) were significant statistically.

The mean duration and standard deviation of presenting complaints in ABPA patients are shown in Table 2.

**Table 2:** Mean duration and standard deviation of presenting complaints in ABPA patients ABPA: Allergic bronchopulmonary aspergillosis

	Mild (n=16)		Moderate (n=14)		Severe (n=37)		F	p-value	Mild vs Moderate	Mild vs Severe	Moderate vs Severe
	Mean	SD	Mean	SD	Mean	SD			p-value	p-value	p-value
Breathlessness duration (Days)	6.56	4.22	9.14	3.34	10.33	4.90	2.216	0.125	0.262	0.043	0.549
Cough duration (Days)	6.25	3.45	11.73	4.58	11.63	5.84	3.529	0.039	0.029	0.015	0.957
Fever duration (Days)	2.67	1.63	2.80	1.79	2.42	1.24	0.140	0.870	0.882	0.737	0.629
Sputum production duration (Days)	5.50	2.74	6.29	2.14	8.06	3.87	1.590	0.222	0.679	0.120	0.250
Passage of brownish or yellowish plugs in sputum duration (Days)	4.00	1.73	5.00	2.40	4.82	2.70	0.177	0.839	0.561	0.610	0.868
Hemoptysis duration (Days)	1.00	.	2.00	1.00	1.71	1.50	0.195	0.826	-		
Chest pain duration (Days)	2.00	1.00	5.00	.	2.57	1.81	1.258	0.335			

The mean values of various laboratory parameters in ABPA categories are shown below in Table 3.

**Table 3:** The mean values of various laboratory parameters in ABPA categories. ABPA: Allergic bronchopulmonary aspergillosis; TLC: Total leukocyte count

	Mild(n=16)		Moderate (n=14)		Severe (n=37)		F	p-value	Mild vs Moderate	Mild vs Severe	Moderate vs Severe
	Median	IQR	Median	IQR	Median	IQR			p-value	p-value	p-value
TLC	11750	10625-13850	18000	14725-20200	16000	12950-18350	10.047	0	0	0	0.226
% Eosinophilic counts	5.3	4.05-7.83	11	6.45-15.25	15.4	10.15-18.40	19.718	0	0.004	0	0.015
Absolute eosinophil counts (cells/μl)	889	737.50-980	1408	1068-1565.25	1220	1107-1411.50	21.789	0	0	0	0.043
Total IgE levels IU/ml	1014	1003.25-1047.50	7909	2803-9436	4500	3250-6331	23.67	0	0	0	0.056
Aspergillus-specific antibodies kUA/l	12.05	15.51-53.95	54.03	3771-7040	41.25	29.28-54.49	29.553	0	0	0	0.062

From our study, there were no appreciable chest X-ray abnormalities in the mild category in comparison to the moderate and severe categories as shown in Table 4 below.

**Table 4:** Chest X-ray abnormalities in the mild category in comparison to the moderate and severe categories.

X-ray findings	ABPA category						Total	Chi-square value	p-value
	Mild (n=16)		Moderate (n=14)		Severe (n=37)				
Normal	16	100%	0	0%	0	0%	16	67	0.000
Fleeting opacity	0	0%	11	79%	31	84%	42	35.431	0.000
Transient consolidation	0	0%	11	79%	31	84%	42	18.283	0.000
Pulmonary infiltrate	0	0%	10	71%	32	86%	42	37.937	0.000
Ring shadows	0	0%	4	29%	15	41%	19	9.036	0.011
Air fluid level	0	0%	0	0%	6	16%	6	5.343	0.069
Central Bronchiectasis	0	0%	14	100%	29	78%	43	39.726	0
Any other	0	0%	1	7%	1	3%	2	1.338	0.512

Table 5 below shows the High Attenuation Mucus (HAM) and centrilobular nodules in 18 patients.

**Table 5:** Presence of high attenuation mucus (HAM) and centrilobular nodules in various categories of ABPA ABPA: Allergic bronchopulmonary aspergillosis

	ABPA category						Total	Chi-square value	p-value
	Mild (n=16)		Moderate (n=14)		Severe (n=37)				
High Attenuation Mucus	0	0%	9	64%	9	24%	18	15.977	0.000
Centrilobular nodules	0	0%	7	50%	25	68%	32	20.474	0.000

The description of the results of spirometry was done to identify the pulmonary functions in various grades of severity. We found out that spirometry was normal in 31% of cases in the mild category. The mixed pattern was due to underlying pulmonary fibrosis in 8% of cases in the severe category. A maximum of 59 patients were found to have an obstruction pattern. On a categorical basis, this pattern was observed in 69% of mild cases, 100% of moderate cases, and 92% of severe cases.

In Table 6, we compared mean values of AEC, Total IgE levels, and Aspergillus-specific IgE in patients with and without central bronchiectasis and these values were significantly higher in patients who had central bronchiectasis.

**Table 6:** Comparison of serological findings in patients with and without central bronchiectasis

Central Bronchiectasis	N		Y		t	p-value	95% Confidence Interval of the Difference	
	Mean	SD	Mean	SD			Lower	Upper
Absolute eosinophil counts (per microlitre)	878.76	170.47	1294.14	273.26	-5.874	0.000	-556.606	-274.142
Total IgE levels (IU/ml)	1176.82	656.16	5453.06	2725.92	-6.375	0.000	-5615.792	-2936.681
Aspergillus-specific antibodies (kUA/l)	12.51	2.44	46.48	18.36	-7.565	0.000	-42.932	-24.999

We also compared the mean values of AEC, total IgE levels, and Aspergillus-specific IgE in patients with and without central bronchiectasis and these values were significantly higher in patients who had HAM as shown in Table 7.

**Table 7:** Comparison of serological findings in patients with and without high attenuation mucus (HAM)

High Attenuation Mucus	N		Y		t	p-value	95% Confidence Interval of the Difference	
	Mean	SD	Mean	SD			Lower	Upper
Absolute eosinophil counts (per microlitre)	1091.49	218.54	1453.50	367.43	-4.944	0.000	-508.241	-215.773
Total IgE levels (IU/ml)	3129.31	2108.20	7740.17	2541.11	-7.503	0.000	-5838.099	-3383.622
Aspergillus-specific antibodies (kUA/l)	30.36	16.74	58.29	21.04	-5.641	0.000	-37.826	-18.046

In Table 8, when we compared the mean values of AEC, total IgE levels, and Aspergillus-specific IgE in patients with and without centrilobular nodules, we found that these values were significantly higher in patients who had centrilobular nodules.

**Table 8:** Comparison of serological findings in patients with and without centrilobular nodules

Centrilobular nodules (CN)	N		Y		T	p-value	95% Confidence Interval of the Difference	
	Mean	SD	Mean	SD			Lower	Upper
Absolute eosinophil counts (per microlitre)	1081.86	353.38	1305.66	199.17	3.153	0.002	82.028	365.573
Total IgE levels (IU/ml)	3203.06	2838.67	5642.25	2721.18	3.583	0.001	1079.661	3798.724
Aspergillus-specific antibodies (kUA/l)	27.80	20.63	48.86	17.39	4.496	0.000	11.707	30.417

## Discussion

Allergic bronchopulmonary aspergillosis (ABPA) is a common fungal infection in uncontrolled asthmatics, cystic fibrosis patients, and immunocompromised patients. Early diagnosis and rapid implementation of proper management are critical to prevent complications and/or disease progression. Diagnosis centres around classic clinical manifestations, radiographic findings, and immunological findings. We did this study to conclude the clinical, microbiological, and radiological profiles of patients with mild-moderate and severe ABPA and to study the radiographic-serological association in ABPA patients.

The age range of all patients with ABPA was 15-65 years. Out of the total of 67 patients enrolled in the study, the maximum number of patients in the mild category, i.e. 31% each, were in the 4th and 5th decade, but the maximum number of patients in the moderate, i.e. 36%, and severe, i.e. 35%, were in 3rd decade. Overall maximum number of patients were either in their third decade or the fifth decade which is similar to studies done by Prasad et al. [9], Subramanian and Natarajan [10], and Kumar [8]. In our study, the overall mean age was found to be 36.42 years. Similar findings were seen in the study by Behera et al. [11], Chakrabarti et al. [12] and Agarwal et al. [5] in which the mean age of 34.3, 36.4, and 33.4 years respectively was documented. However, in the mild category, the mean age group was 43.25 years, whereas in the moderate mean age was 31.5 and in the severe category, it was 35.3 years. In a study done by Kumar [8] mean ages of the mild, moderate and severe groups were 30, 22.5 and 36 years, respectively.

All the patients that were included in this study had a previous history of asthma which was similar to the findings made by Agarwal et al. [12]. However, Behera et al. [11] reported a 94% incidence of asthma in their study and Chakrabarti et al. [13] gave a 90% incidence of asthma in their study. The mean duration of asthma in our study was 13.6 years. In a study done by Agarwal et al. [14], based on different grades of severity median duration of asthma was 6 years in mild with an IQR range of (5-12) years, similarly in the moderate category 7 years (4-15) and 5 years (3-10) in the severe category. In this study mean duration of asthma was 8.69 ± 7.68 years in the mild category, 12.5 ± 5.17 years in the moderate and 16.14 ± 6.22 years in the severe category. Glancy et al. reported 11 cases of ABPA without asthma [15].

From Table 2, we found that cough was the main complaint in the moderate and severe categories as it was present in 79% and 65% of cases respectively whereas, in the mild category, the cough was present in only 50% of cases. Prasad et al. [9] also found in their study that cough was present in 50% of cases. Expectoration was documented in 38% of cases of mild, 50% of moderate and 49% of the severe category. Similarly, expectoration of brownish and yellowish sputum plugs was present in 64% of cases of the moderate category. Mucopurulent expectoration has been documented and described by Chakrabarti et al. [13] and Prasad et al. [9] in 68.5 and 57.1% of cases. Kumar [8] documented the expectoration of yellowish plugs as 33% in both mild and moderate categories whereas it was 50% in the severe category. Agarwal et al. [14] documented brownish-yellowish plugs in 39.7% of cases. History of haemoptysis was present in 6% of mild, 21% of moderate and 19% of severe category patients with an overall percentage of 16%, but in a study done by Kumar [8], haemoptysis was present in 33% of cases of mild and 50% of patients in the severe category. Agarwal et al. [14] reported that 36.8% of their patients had a history of haemoptysis.

Total IgE level is one of the major criteria given by Rosenberg et al. to diagnose ABPA [6]. According to the ISHAM working group, the cut-off level of total IgE is > 1000 IU/ml for ABPA [14]. In our study, total IgE levels were done in all patients and the median value of IgE in the mild category was 1014 IU/ml (1003.25-1047.50), but it was significantly higher in the moderate group 7909 IU/ml (2803-9436) in comparison to the severe group where it was 4500 IU/ml (3771-7040) (Table 3). In a study done by Kumar et al. [8], values of total IgE were 597 ± 330.22 IU/ml, 2571 ± 1492.14 IU/ml, and 3435 ± 3948.7 IU/ml. We observed that values of total IgE levels were significantly more in the moderate category, especially in those patients who had HAM positive. Similarly, AEC (> 1000 cells/μL) is also one of the major diagnostic criteria which is also used as a diagnostic indicator in a patient with asthma. In the present study, median value of AEC in the mild group was 889 cells/μL whereas it was 1408 cells/μL in the moderate and 1220 cells/μL in the severe group which again showed that AEC was higher in the moderate category.

Aspergillus-specific antibodies were 11.97 ± 1.01 kUA/L in mild, 52.89 ± 19.38 kUA/L in moderate and 43.37 ± 17.76 kUA/L in the severe category in the present study, however, in a study done by Agarwal et al. [14] mean Aspergillus-specific IgE levels were 2.1 (0.95-9.5) kUA/L in mild, 5.6 (1.9-17.9) kUA/L in moderate and 7.8 (4.7-23.7) kUA/L in the severe category. We observed that values of AEC, total IgE and Aspergillus-specific IgE levels were higher in each category in our study compared to studies by Kumar [8] and Agarwal et al. [14] and moreover we found that these serological parameters were comparatively higher in the moderate group.

**Figure 2:**
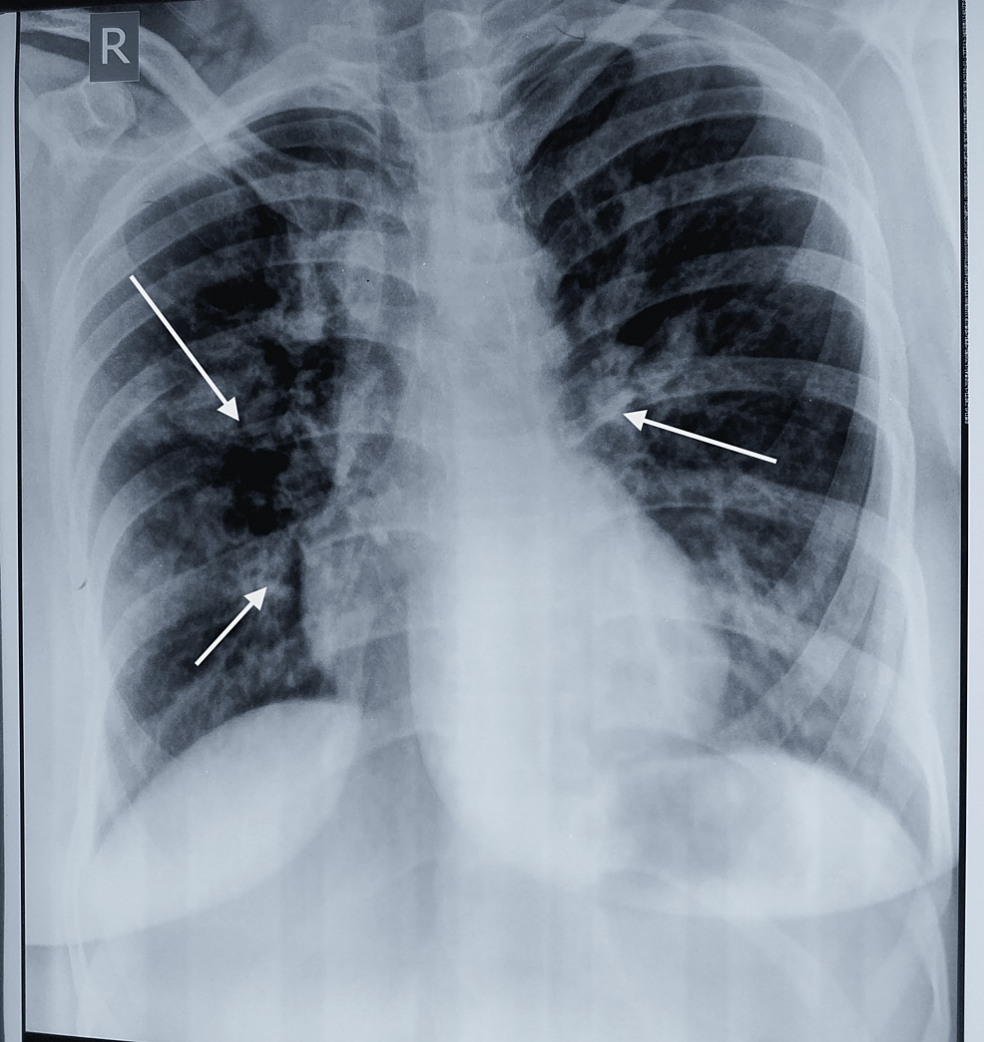
Chest X-ray of ABPA patient with central infiltrates and fleeting opacities (marked with white arrows). ABPA: Allergic bronchopulmonary aspergillosis

ABPA is also called “PICTURESQUE” disease because of the wide variation seen in the appearance of chest radiography. These images are either transient or permanent. In the seminal description of ABPA, Hinson et al. [16] found that fleeting opacities are characteristic features of this disease. In our study, fleeting opacities were seen in 67% of patients, on category-wise distribution fleeting opacities were seen in 84% cases of severe cases and 79% of cases in the moderate category (Figure 2). In the present study, central bronchiectasis and pulmonary infiltrates were present in the majority of patients (64%). Other studies showed abnormal chest X-ray findings in which pulmonary infiltrates were the most common finding and distributed mainly in upper zones [13]. There were no appreciable X-ray abnormalities in the mild category in comparison to the moderate and severe categories. Regarding HRCT, computed tomography (CT) was normal in 24% of cases of the mild category which was similar to the study done by Kaur and Sudan [17] who documented 22% of cases of normal CT in their study.

**Figure 3:**
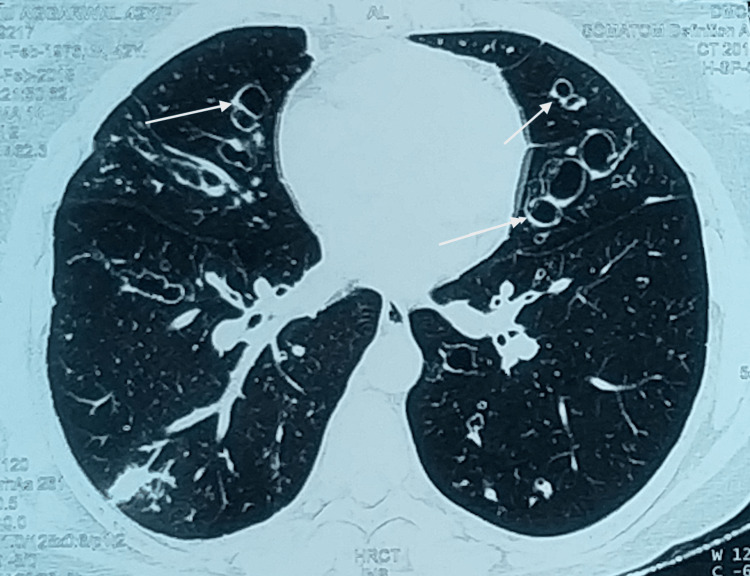
Central bronchiectasis of ABPA patient with signet rings (marked with white arrows) ABPA: Allergic bronchopulmonary aspergillosis

Central bronchiectasis (Figure 3) was seen in 76.1% of cases of ABPA which was similar to studies done by Behera et al. [11], Chakrabarti et al. [13], and Agarwal et al. [14] which reported incidence as 71%, 69% and 76.1%, respectively. A study by Mitchell et al. [18] documented that 89% of patients with ABPA had bronchiectasis involving several lobes. In a study by Kaur and Sudan [17], 78% of patients demonstrated central bronchiectasis on HRCT with a predilection for upper and middle lobes. Peripheral bronchiectasis was also seen in 23.8% of cases with similar findings recorded in a study by Neeld et al. [19] in which 25% had peripheral bronchiectasis. The study done by Agarwal et al. [20] showed evidence of peripheral bronchiectasis were 7.1% and 33.1%.

Mediastinal lymphadenopathy was seen in 43% of cases which were not comparable due to the lack of literature on lymphadenopathy but there were case reports by Hachiya et al. [21] and Shah et al. [22] in which they reported hilar lymphadenopathy which regressed after administration of steroids to patients.

Mucus plugs which appeared hyperdense on CT (attenuation more than 70 Hounsfield units) were labelled as high attenuation mucus (HAM) [14]. In this study, 26.8% of patients had high attenuation mucus present in HRCT. Another study by Agarwal et al. [14] quotes 22% evidence of mucus which was denser than adjoining paraspinal muscle (HAM); they also proposed that ABPA should be classified on the basis of the presence of HAM, that is ABPA-CB-HAM, as a severe group because it was more related to serological findings than previous classification by Kumar [8] which relied on other radiological features. According to Agarwal et al., ABPA-CB-ORF represented the burnt-out phase of the disease and hence should not be classified as severe [14].

Centrilobular nodules which lead to a characteristic tree in bud appearance were seen in 48% of cases of ABPA which was marginally higher in comparison to a study done by Agarwal et al. [20] who documented centrilobular nodules in 31.7% of cases. However, Kaur and Sudan [17] gave evidence of 86% in their study.

In summary, ABPA is a complex hypersensitivity response to fungal antigens, either mycelia or spores, most commonly seen in patients with bronchial asthma. The overall mean age of presentation is the third decade but in the mild category, the presentation can be in the fourth or fifth decade also. The disease has no gender predisposition, however, in category-wise distribution majority of mild and severe cases were males and most of the moderate disease cases were found to be females. All patients included in the study had positive type I hypersensitivity response, while type III response was present in almost half of the patients only. Fleeting opacities and pulmonary infiltrates were the most common findings on chest X-rays but ABPA can still be present with no specific radiological abnormality. On HRCT, central bronchiectasis was the commonest finding seen in most of the patients. However, it was seen that patients with HAM and centrilobular nodules had more severe clinical symptoms than the rest of the patients.

The majority of patients showed an obstructive pattern on spirometry and moderate to severe obstruction was the commonest pattern observed among patients who had an obstructive pattern on spirometry. Also, all three patients with the mixed pattern on spirometry had severe disease.

Serological analysis revealed that patients in the moderate category had a higher level of AEC, total IgE, and Aspergillus-specific IgE antibodies, especially in patients who had either HAM or centrilobular nodules on their HRCT scan.

## Conclusions

In summary, ABPA is a complex hypersensitivity response to fungal antigens, either mycelia or spores, most commonly seen in patients with bronchial asthma. From our study, we concluded that the clinical, microbiological and radiological features were observed due to more active inflammation in patients with HAM or centrilobular nodules in comparison to patients classified as having a severe disease based on other radiological findings (ORF) which are sequelae of chronic inflammation and not disease activity itself.

Besides, in a previous landmark study done by Agarwal et al., their inference was right to have alternate criteria for the classification of ABPA which was not based on the sequelae of chronic inflammatory changes in the lungs.
